# BiotaPredictor: A machine learning framework for predicting drug depletion by the human gut microbiota

**DOI:** 10.1080/19490976.2026.2743953

**Published:** 2026-10-15

**Authors:** Benyamin Naranjani, Laura E. McCoubrey, Anders Borde, Gustaf Hulthe, Mats Josefson, Susanna Abrahmsén-Alami, Bertil Abrahamsson, Abdul W. Basit

**Affiliations:** a Department of Pharmacy, Uppsala Biomedical Center, Uppsala University, Uppsala, Sweden; b UCL School of Pharmacy, University College London, London, UK; c Global Product Development, Pharmaceutical Technology & Development, Operations, AstraZeneca, Gothenburg, Sweden

**Keywords:** Oral drug delivery, gastrointestinal microbiome, biotransformation of medications, drug metabolism and bioaccumulation, artificial intelligence, pharmacomicrobiomics

## Abstract

The gut microbiota can profoundly influence the performance of oral medicines by metabolizing or bioaccumulating drugs, thereby reducing systemic exposure and contributing to variable patient responses. Despite growing recognition of these interactions, microbiota-driven drug depletion remains insufficiently addressed during early development. Here, we present BiotaPredictor, a freely accessible web-based machine learning platform that predicts the likelihood of drug depletion by human intestinal microbiota from molecular structure. We compiled two curated datasets containing microbial depletion outcomes: (i) a consistently annotated AstraZeneca dataset (158 compounds) and (ii) an expanded integrated dataset incorporating published evidence (522 compounds). Multiple molecular descriptors—including physicochemical properties, 3D conformational features, and fingerprint-based encodings—were evaluated across a suite of classification algorithms. A logistic regression model trained on the AstraZeneca dataset using physicochemical parameters achieved the highest performance, correctly classifying all compounds in an external validation study (3 depleted, 9 stable). BiotaPredictor enables early triage of compounds susceptible to microbiota-mediated loss and can inform lead optimization, formulation strategy, and pharmacokinetic prediction. Integrating microbiota liability assessment into development pipelines will contribute to safer, more efficacious drug products and support precision delivery of orally administered therapeutics.

## Graphical abstract

**Figure:**
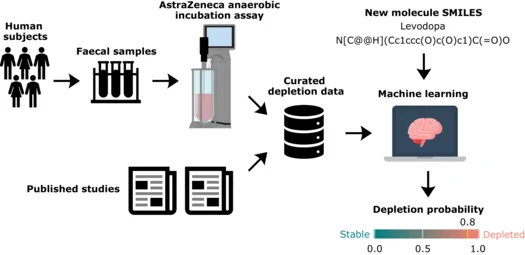

## 1. Introduction

Stability of orally administered drugs within the gastrointestinal (GI) tract is a critical determinant of oral drug delivery performance, bioavailability, therapeutic efficacy, and patient safety. A growing body of evidence demonstrates that the gut microbiota can alter drug exposure through metabolism or bioaccumulation, processes collectively referred to here as microbial drug depletion, ultimately reducing the amount of active drug available for absorption or systemic action and contributing to variable patient responses.^1^ ^,^ ^2^ This is particularly relevant in the distal intestine, where microbial abundance and enzymatic diversity increase substantially.^3^ Compounds that reach the colon in significant amounts—including poorly soluble or poorly permeable drugs, incompletely absorbed molecules, and drugs formulated for modified release (e.g., extended release or delayed release formulations)—may therefore be particularly susceptible to microbiota-mediated degradation.

Despite these implications, microbial drug depletion remains rarely assessed during oral drug candidate selection, biopharmaceutics evaluation, or formulation design. As a result, microbiota-related instability may only become apparent during late-stage development, clinical evaluation, or post-approval use, when reformulation or compound redesign becomes more costly and difficult. Early identification of compounds susceptible to intestinal microbial depletion would support formulation optimization, improve interpretation of pharmacokinetic variability, and guide the development of more robust oral drug products.

For oral and modified-release formulation development, structure-based molecular modeling and machine learning provide a practical route to screen compounds for microbiota-mediated depletion before extensive experimental testing. Three-dimensional molecular descriptors capture spatial configuration, molecular shape, and functional group orientation, providing information relevant to how drugs interact with microbial enzymes, binding sites, or cellular uptake processes.^4-6^ When combined with physicochemical descriptors and structural fingerprints, these molecular representations support prediction of metabolism, bioaccumulation, and stability under anaerobic intestinal conditions.

Here, we developed and benchmarked machine learning classifiers to predict microbiota-mediated drug depletion as an early screening approach for oral drug delivery and formulation development. The novelty of this work lies in linking standardized anaerobic microbiota stability data with molecular descriptors to identify compounds susceptible to intestinal microbial loss before extensive experimental testing. This is particularly relevant to the development of extended-release and colon-targeted formulations, where prolonged or distal intestinal exposure increases the likelihood of microbiota-mediated drug loss and highlights the importance of considering these interactions during early-stage screening.^7^ More broadly, this approach may support the development of next-generation microbiome-informed drug delivery systems, in which microbiota-associated processes are deliberately considered as determinants of drug release, biotransformation, and localization.^8^ Models were trained using two complementary datasets: (i) a controlled and consistently labeled AstraZeneca dataset generated under standardized anaerobic fecal incubation conditions, and (ii) a larger integrated dataset combining the AstraZeneca data with peer-reviewed evidence from the literature. We further performed anaerobic depletion experiments to provide an external assessment of model predictions for selected compounds. The final models were implemented in BiotaPredictor, an openly available web resource (https://biotapredictor.onrender.com/) designed to support routine assessment of microbiota-mediated intestinal drug loss during pharmaceutical research and development. This framework provides a practical route to incorporate gut microbiota stability into early candidate triage, controlled and extended-release formulation optimization, and biopharmaceutics risk assessment. Overall, this work advances the integration of pharmacomicrobiomics into oral drug delivery by supporting earlier identification of microbiota-sensitive compounds before late-stage formulation or clinical evaluation.

## 2. Materials and methods

### 2.1. Data curation

#### 2.1.1. AstraZeneca dataset

Our study used a core AstraZeneca (AZ) dataset comprising 158 compounds characterized in vitro for depletion by human fecal microbiota. Human fecal material used to generate the AstraZeneca dataset was obtained either from in-house AstraZeneca slurry preparation approved by the Regional Ethical Review Board in Gothenburg, University of Gothenburg, Sweden, under diary number 462-06, or as ready-to-use human intestinal microbiota slurry from the Culture Collection of Switzerland (CCOS), with supplier documentation confirming voluntary donation, third-party research and commercial use, and anonymized donor data handling. The study was conducted in accordance with relevant ethical guidelines and the principles of the Declaration of Helsinki. The experimental setup has been described previously and consisted of a static anaerobic in vitro incubation system containing mixed human fecal microbial communities.^9^ Briefly, human fecal material from healthy volunteers was processed under anaerobic conditions and homogenized with physiological saline to generate a fecal homogenate representative of human colonic microbial activity. Test compounds were incubated with the homogenate at 37 °C, and drug remaining over time was quantified from collected samples to determine depletion profiles, half-lives, and percentage remaining. All experimental work involving human fecal material was conducted in accordance with applicable institutional biosafety and laboratory health and safety procedures. The AZ dataset included investigational and established drugs, including zonisamide, rofleponide, omeprazole, metronidazole, and budesonide, spanning diverse molecular structures and pharmacological classes. The full compound list and detailed class-level breakdown of internal AZ compounds cannot be disclosed because of confidentiality restrictions.

For model development, compounds were labeled as depleted when they met either of the following AZ in vitro stability criteria: a half-life of less than 250 min or a decrease of more than 10% in drug remaining after 1 h of incubation with human fecal microbiota (Figure 1a). Previous work showed that the percentage of drug remaining in this AZ in vitro system correlates with the fraction absorbed from the human colon,^9^ supporting the biopharmaceutics relevance of these thresholds. These thresholds were used to identify intrinsic susceptibility to microbiota-mediated depletion under intestinal microbial exposure, rather than to predict if depletion has clinically significant impact on pharmacokinetics. For rapidly absorbed immediate-release drugs, the pharmacokinetic relevance of depletion is expected to be lower if limited drug reaches the distal intestine, whereas the risk is greater for compounds with distal intestinal exposure, including controlled-release, extended-release, and enteric-coated products. Using these criteria, 36 of the 158 compounds were labeled as depleted by the microbiota, while the remaining compounds were labeled as stable.

To provide an external assessment of model predictions, 12 compounds were tested in the AZ in vitro incubation system: ozanimod, methotrexate, tacrolimus, and nine internal AZ compounds. Each experiment was performed in triplicate. A compound was classified as experimentally stable when the mean amount of drug remaining across triplicates stayed above the established AZ stability threshold after 2 h of incubation. These compounds were not used for model training, feature selection, or hyperparameter tuning. For models trained on the Integrated dataset, one compound overlapping with the training data was excluded from the external evaluation.

**Figure 1.**
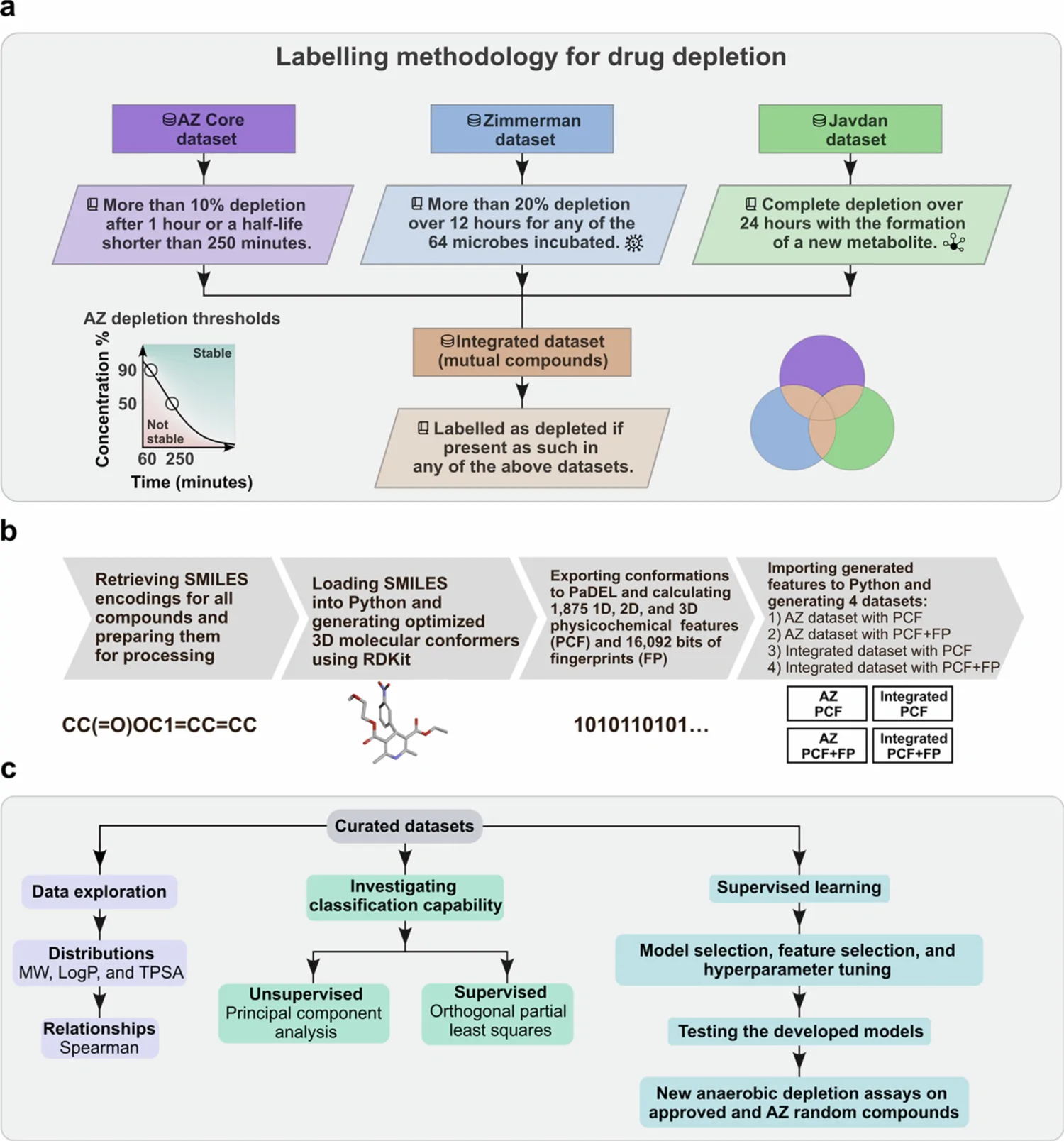
Dataset labeling and modeling workflow. **a** Labeling strategy for microbial drug depletion across the AZ dataset and literature-derived data, including the Zimmermann^10^ and Javdan^11^ datasets. Literature data were labeled using equivalent depletion criteria where compatible with the reported data format. **b** Molecular feature generation workflow, including SMILES extraction, RDKit-based conformer generation and energy minimization, and calculation of 1875 molecular descriptors and 16,092 fingerprint bits using PaDEL. **c** Overall modeling pipeline, including data exploration, feature generation, supervised learning, feature selection, hyperparameter tuning, and model evaluation.

#### 2.1.2. Integrated dataset

To expand the chemical and experimental coverage beyond the AZ dataset, an Integrated dataset was generated by combining the AZ dataset (*n* = 158 compounds) with additional human microbiota-related data from public repositories, literature reviews, and experimental studies (*n* = 522 compounds).^10^ ^,^ ^11^ Before duplicate removal, 10 literature-derived compounds overlapped with compounds in the AZ dataset; these duplicate entries were harmonized during dataset integration, and compounds were labeled as depleted if they were classified as depleted in any source dataset. The AZ dataset was intentionally included as the internally consistent core component of the Integrated dataset; therefore, the Integrated dataset was not used as an independent validation set for the AZ models. The additional data included small molecule stability measurements in the presence of gut bacterial isolates, human fecal microbiota, or human intestinal fluid. Animal data were excluded because of known differences between animal and human gut microbiota composition.

Because the additional data were derived from heterogeneous experimental sources, stability labels were harmonized using criteria adapted to the available data format. Half-life values and 1 h depletion measurements were not consistently available across the literature-derived compounds. Therefore, for the additional data, compounds showing less than 20% depletion after 12 h of incubation with microbiota were labeled as stable, whereas compounds showing 20% or greater depletion were labeled as depleted (Figure 1a). Using this criterion, 216 of the 522 additional compounds were labeled as depleted. After combining the AZ dataset with the additional data, the final Integrated dataset contained 680 compounds, of which 252 were labeled as depleted.

### 2.2. Molecular descriptor and fingerprint calculation

Molecular descriptors and fingerprints were generated to represent structural and physicochemical properties relevant to microbiota-mediated drug depletion. Because molecular geometry influences interactions with microbial enzymes, binding sites, and cellular uptake processes, three-dimensional descriptors were included alongside one- and two-dimensional descriptors. For each compound, canonical simplified molecular input line entry system (SMILES) strings were used to generate 50 conformers with RDKit (Figure 1b).

Conformer generation was performed in RDKit using the EmbedMultipleConfs function, with useExpTorsionAnglePrefs = True and useBasicKnowledge = True to incorporate experimental torsion angle preferences and general chemical knowledge. For each conformer, energy minimization was performed using either the Merck Molecular Force Field (MMFF) or the Universal Force Field (UFF), depending on parameter availability. The lowest energy conformer was selected as a standardized three-dimensional molecular representation for descriptor calculation. These conformers were then processed using PaDEL Descriptor to compute 431 three-dimensional molecular descriptors capturing structural characteristics relevant to microbiota-mediated depletion.^12^

In addition to the 431 three-dimensional descriptors, 1444 one-dimensional (1D) and two-dimensional (2D) molecular descriptors were computed using PaDEL. These descriptors represented chemical composition, connectivity, topology, molecular weight, polarity, and other physicochemical properties relevant to compound reactivity and microbiota-mediated stability.

Molecular fingerprints were also calculated using PaDEL, comprising 12 fingerprint types and 16,092 bits. These fingerprints encoded structural and substructural patterns, including atom type electrotopological state descriptors, Crippen logP and molar refractivity descriptors, extended topochemical atom descriptors, Klekota Roth substructures, Laggner substructure patterns, and binary fingerprints. Fingerprints were included to capture discrete molecular motifs that may not be fully represented by continuous physicochemical descriptors, while maintaining a reproducible and predefined PaDEL-based feature set.

### 2.3. Model selection and evaluation

Stable and depleted classifications were used as binary labels for supervised model training and evaluation. Five machine learning algorithms were evaluated to capture both linear and nonlinear relationships between molecular features and microbiota-mediated depletion: logistic regression, balanced random forest, support vector machine, LightGBM, and XGBoost (Figure 1c). In the figures, LightGBM is abbreviated as GBM for compact labeling. These models were selected because the datasets were imbalanced, chemically diverse, and represented by high-dimensional physicochemical descriptors and molecular fingerprints. Tree-based ensemble methods, including LightGBM and XGBoost, were included because they handle nonlinear feature interactions and sparse molecular fingerprints effectively.^13-16^ The model set also reflects the broader need to evaluate computational approaches for drug metabolism and interaction-related risks during development.^17^ Class imbalance was addressed during model development using algorithm-specific strategies, including balanced sampling for balanced random forest and class weighting where applicable. For XGBoost, the scale_pos_weight parameter was tuned to improve representation of the depleted class.^18^

Balanced random forest (BRF) was included to address class imbalance through balanced bootstrap sampling while retaining the interpretability of tree-based ensemble models.^19^ Feature importance estimates from BRF were used to support interpretation of molecular descriptors associated with microbial depletion.

Support vector machine (SVM) with a radial basis function (RBF) kernel was included to capture nonlinear decision boundaries in high-dimensional molecular feature space. Class-specific weighting was applied through class_weight in scikit-learn to improve representation of the depleted class during training.^20^ Logistic regression was included as an interpretable linear classifier that provides probabilistic outputs and supports assessment of relationships between selected molecular descriptors and microbial depletion.

All model development and cross-validation analyzes were performed separately for each dataset and feature scenario. Preprocessing steps, including imputation and scaling, were fitted within the training folds and then applied to the corresponding held-out folds to minimize information leakage during cross-validation.

Model performance was assessed using balanced accuracy, F1 score, and area under the precision-recall curve (AUPRC), with depleted compounds defined as the positive class. These metrics were selected to evaluate classification performance in imbalanced datasets by capturing sensitivity to both stable and depleted classes, the balance between precision and recall, and positive class performance across classification thresholds. Results were reported across stratified cross-validation folds. The computational workflow was implemented using scikit-learn (1.4.2), imbalanced-learn (0.12.2), RDKit (2023.9.5), XGBoost (1.7.6), and LightGBM (4.3.0).

### 2.4. Feature selection and hyperparameter tuning

Recursive feature elimination (RFE) was applied to reduce model complexity, limit overfitting, and identify informative molecular descriptors before final model training. RFE was selected because it provides an iterative wrapper-based approach for feature selection and is well suited to high-dimensional classification problems with limited sample size.^21^ To further reduce overfitting risk, the number of selected features was constrained to no more than half the number of compounds in each dataset.

Model hyperparameters were optimized using grid search with stratified k-fold cross-validation. Stratification preserved the stable-to-depleted class ratio across folds, reducing bias introduced by class imbalance during model selection.

For logistic regression, solver and regularization settings were optimized using scikit-learn, including liblinear and saga solvers with L1 and L2 penalties.^22^ Final configurations for the physicochemical and combined feature models are provided in Table S1.

For BRF, we tuned the number of estimators (n_estimators), splitting criterion (entropy), and feature consideration at each split (max_features). Class imbalance was handled by BRF's balanced bootstrap sampling (sampling_strategy = ‘all’); no additional class weighting was used in the final models (Table S2).

XGBoost (v1.7.6) and LightGBM (v4.3.0) were evaluated using their default learning rates of 0.3 and 0.1, respectively. Regularization and early stopping were applied where applicable to limit overfitting. For support vector machine models, the regularization parameter, kernel coefficient, and class weighting settings were explored, with class weighting used to improve representation of the depleted class during training.

### 2.5. Correlation analysis

Spearman correlation analysis was used to examine associations between molecular features and binary microbial depletion labels across four modeling scenarios: AZ dataset with physicochemical descriptors, AZ dataset with physicochemical descriptors and fingerprints, Integrated dataset with physicochemical descriptors, and Integrated dataset with physicochemical descriptors and fingerprints. Spearman's rank correlation was selected instead of Pearson’s correlation to capture monotonic relationships without assuming linearity.^23^ This analysis identified physicochemical descriptors and substructural features associated with microbial depletion.

### 2.6. Principal component analysis

Unsupervised principal component analysis (PCA) was applied to visualize compound distribution and assess whether stable and depleted compounds showed clustering in reduced molecular feature space. PCA was performed separately for the AZ and Integrated datasets using physicochemical descriptors alone and physicochemical descriptors combined with fingerprints. The resulting score plots were used to evaluate whether depletion classes formed discernible patterns before supervised model development.

### 2.7. Orthogonal partial least squares analysis

Orthogonal partial least squares (OPLS) analysis was applied to examine supervised class separation between stable and depleted compounds across the four modeling scenarios: AZ and Integrated datasets, with and without fingerprints. OPLS separates predictive variation associated with the response variable from orthogonal variation unrelated to class assignment. The analysis was performed using SIMCA 18.0.1. For each model, the first predictive component, t[1], was used to assess whether compounds clustered according to microbial depletion class.

Score plots were colored according to the binary depletion label. R2X values were extracted to quantify the proportion of variance explained by the predictive component, R2X[1], and the first orthogonal component, R2Xo[1]. These values, together with visual separation along t[1], were used to qualitatively assess whether the feature space contained a class-related structure before supervised classification.

### 2.8. Statistical analysis of model performance

Statistical comparisons of model performance were performed using balanced accuracy, F1 score, and AUPRC values obtained from stratified 10-fold cross-validation. One-way ANOVA followed by Tukey post hoc testing was used to compare multiple models within the same dataset and feature scenario. For pairwise comparisons, including comparisons before and after feature selection or hyperparameter tuning, Welch's t-test was used to account for unequal variances. Differences were considered statistically significant at *p* < 0.05. In figures, significance levels are indicated as follows: *p* < 0.05 (*), *p* < 0.01 (**), *p* < 0.001 (***), *p* < 0.0001 (****); ns denotes non-significant differences.

## 3. Results

### 3.1. Conformer generation

To assess whether the conformer generation workflow produced chemically reasonable three-dimensional structures for descriptor calculation, we compared an experimentally resolved nimodipine structure from the Protein Data Bank (PDB ID: 5KMF, Figure S1a) with the lowest energy nimodipine conformer generated by force field-based optimization (Figure S1b, c). The generated conformer showed reasonable agreement in overall molecular geometry and the orientation of key functional groups. More computationally intensive quantum chemical methods, such as B3LYP-D3, provide higher-level conformational refinement, but force field-based optimization was selected to enable scalable conformer generation across the full compound set. This balance between chemical plausibility and computational efficiency was necessary for descriptor generation, predictive modeling, and web-based implementation.

### 3.2. Data overview and correlations

#### 3.2.1. The relationship of single features with compounds' microbial stability

We first examined the distribution of key physicochemical properties in the AZ and Integrated datasets to evaluate the chemical space represented by stable and depleted compounds. This analysis focused on molecular weight, logP, and topological polar surface area (TPSA), which are directly relevant to oral drug delivery, intestinal exposure, permeability, and microbiota-mediated stability. Because depleted compounds represented the minority class, these distributions were also used to assess whether depletion outcomes were distributed across the chemical feature space rather than confined to a narrow subset of compounds.

In the AZ dataset, molecular weight, logP, and topological polar surface area (TPSA) showed distinct distribution patterns between stable and depleted compounds. Most compounds had molecular weights between 250 and 500 g/mol, and depleted compounds were more frequently observed at lower molecular weights compared to stable compounds (Figure 2a). This pattern indicates that smaller molecules were more susceptible to microbial depletion under the tested anaerobic conditions. Depleted compounds also clustered mainly at logP values between 0 and 2, suggesting greater depletion among relatively polar compounds in the AZ dataset (Figure 2b). TPSA distributions further showed that depleted compounds were predominantly observed above 100 Å^2^, indicating an association between higher polar surface area and microbial depletion (Figure 2c).

The Integrated dataset showed broadly similar but more dispersed distribution patterns, reflecting its larger size and greater chemical diversity. Depleted compounds were again more frequent at lower molecular weights, supporting the association between smaller molecular size and microbiota-mediated depletion across datasets (Figure 2d). In contrast to the AZ dataset, depleted compounds in the Integrated dataset extended across a wider logP range, with enrichment at higher logP values in some bins (Figure 2e). TPSA also showed broader behavior, with depleted compounds spanning values below and above 100 Å^2^ (Figure 2f). These findings indicate that molecular weight showed the most consistent relationship with depletion across datasets, whereas lipophilicity and polar surface area were more dataset-dependent. This supports the use of multiple molecular descriptors rather than relying on single physicochemical properties to predict microbiota-mediated drug stability.

**Figure 2.**
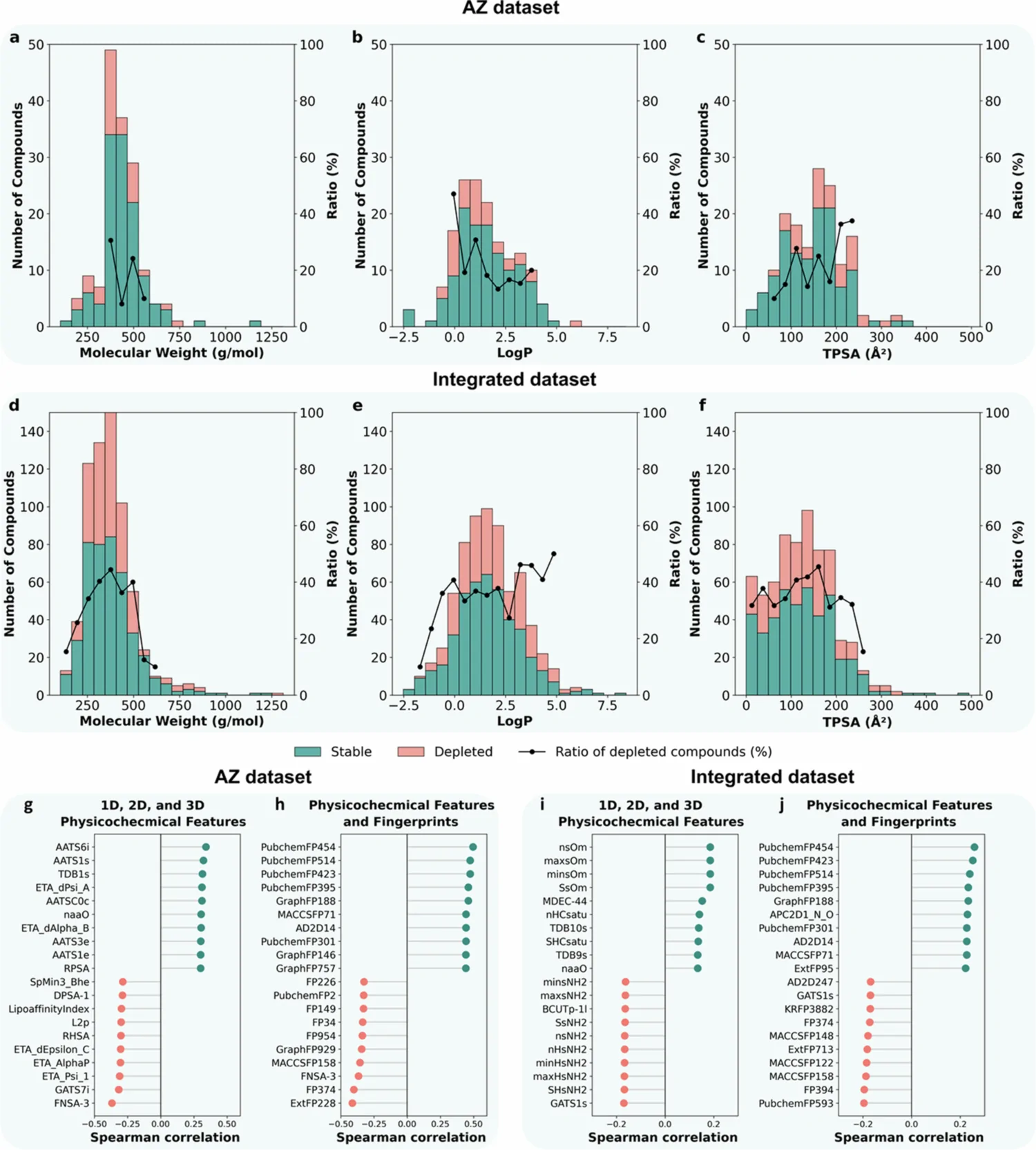
Physicochemical property distributions and feature correlations in the AZ and Integrated datasets. Panels **a–f** show the distributions of molecular weight (**a, d**), logP (**b, e**), and topological polar surface area (TPSA; **c, f**) across stable and depleted compounds in the AZ dataset (**a–c**) and Integrated dataset (**d–f**). Solid black lines with circles indicate the percentage of depleted compounds in bins containing more than 10 compounds. Panels **g–j** show Spearman correlation coefficients for the ten most positively and negatively correlated physicochemical descriptors and fingerprint features associated with microbial depletion in the AZ dataset (**g, h**) and Integrated dataset (**i, j**).

The chemical space represented in both datasets was broadly consistent with marketed oral drug space. Most marketed oral drugs have molecular weights in the range of 300 to 500 g/mol, aligning with the distributions observed in the AZ and Integrated datasets.^24^ ^,^ ^25^ This supports the relevance of the datasets for oral drug delivery applications. Within this drug-like space, lower molecular weight compounds were more frequently depleted by the microbiota, particularly in the AZ dataset. Although this relationship is not a dominant trend in general drug-like property analyzes, it is consistent with the possibility that smaller compounds have greater accessibility to microbial uptake or enzymatic transformation. Importantly, microbiota-mediated depletion is relevant beyond drugs designed for colonic delivery. From a biopharmaceutics and safety perspective, depletion is also relevant for drugs intended for upper gastrointestinal absorption when unabsorbed fractions reach the distal intestine.^26^ ^,^ ^27^ These observations support early evaluation of microbiota stability during oral drug development, including for formulations not primarily designed to target the large intestine.

Because depleted compounds represented the minority class, we assessed whether they were distributed across the physicochemical feature space rather than confined to narrow property ranges. Depleted compounds were observed across multiple descriptor ranges in both datasets, supporting the suitability of the data for supervised classification. This distribution provided the models with chemically diverse examples of microbiota-mediated depletion, which is important for learning patterns that extend beyond a single compound class.

##### 3.2.1.1. Correlations of drug properties with depletion

Spearman correlation analysis was used to examine associations between individual molecular features and microbial depletion across the AZ and Integrated datasets, with and without molecular fingerprints. Across all modeling scenarios, the AZ dataset showed stronger correlations between molecular features and microbial depletion than the Integrated dataset (Figure 2g and h). This likely reflects the greater experimental consistency and narrower chemical space of the AZ dataset, which reduces noise and makes feature outcome relationships more discernible. Consistent with this interpretation, well-curated datasets focused on defined chemical spaces often yield clearer structure-activity relationships.^28^

Inclusion of molecular fingerprints increased the magnitude and number of feature depletion correlations, particularly in the larger and more chemically diverse Integrated dataset (Figure 2i and j). This partly reflects the larger number of input features but also highlights the ability of fingerprints to encode substructural motifs associated with compound microbiota interactions. Such motifs reveal relationships that are not captured by general physicochemical descriptors alone.^29^

Notably, PubChemFP454, PubChemFP514, and PubChemFP423 were consistently among the top correlated fingerprint features in both datasets when fingerprints were included (Figure 2h and j). These features represent substructural motifs with plausible links to microbial depletion. PubChemFP454 corresponds to atom neighborhoods containing the N(─C)(═O) substructure, commonly found in amide groups. Amide-containing compounds undergo hydrolysis in the presence of intestinal microbial enzymes, supporting a potential association with reduced stability.^30^ PubChemFP514 captures the O─N─C─C SMARTS pattern, associated with nitroso or nitramine functionalities. These groups are susceptible to anaerobic reduction or nitrosation reactions mediated by microbial enzymes in the colon.^31^ They are also recognized in ICH M7 guidance as structural alerts relevant to DNA-reactive mutagenic impurities, indicating that such motifs may have implications beyond stability alone.^32^ PubChemFP423 is linked to the N═O substructure, characteristic of nitro groups, which are frequently reduced under anaerobic microbial conditions and may therefore contribute to compound depletion.^33^

The recurrence of these fingerprint features across datasets supports the relevance of specific substructural motifs in microbiota-mediated drug instability. Their association with hydrolysis and reductive metabolism indicates that such motifs provide useful predictive markers for identifying compounds susceptible to intestinal microbial depletion.

Together, these findings show that physicochemical descriptors and molecular fingerprints provide complementary information for characterizing susceptibility to microbiota-mediated depletion. Physicochemical descriptors were more informative in the controlled and narrower AZ dataset, whereas the chemically diverse Integrated dataset benefited from substructure-based fingerprint features.

### 3.3. Investigation of classification capability

#### 3.3.1. Unsupervised principal component analysis

Principal component analysis (PCA) was used to visualize the molecular feature space and assess whether stable and depleted compounds separated without supervision. In the AZ dataset, PCA showed no clear separation between stable and depleted compounds (Figure S2a, b). The first two principal components explained more variance when only physicochemical descriptors were used (PC1: 30.58%, PC2: 10.51%) than when fingerprints were included (PC1: 9.12%, PC2: 5.23%), reflecting the increased dimensionality introduced by fingerprint features.

A similar pattern was observed in the Integrated dataset (Figure S2c, d). The first two principal components explained more variance when physicochemical descriptors were used alone (PC1: 30.24%, PC2: 8.52%) than when fingerprints were included (PC1: 7.73%, PC2: 4.06%). Visual separation between stable and depleted compounds remained weak in both feature spaces, indicating that depletion-related structure was not clearly captured by unsupervised linear projection alone.

#### 3.3.2. Supervised orthogonal partial least squares analysis

Supervised orthogonal partial least squares (OPLS) analysis was applied to assess whether depletion-related class structure became clearer when the binary stability label was included in the projection. OPLS was performed for the AZ and Integrated datasets using physicochemical descriptors alone and physicochemical descriptors combined with fingerprints (Figure 3).

**Figure 3.**
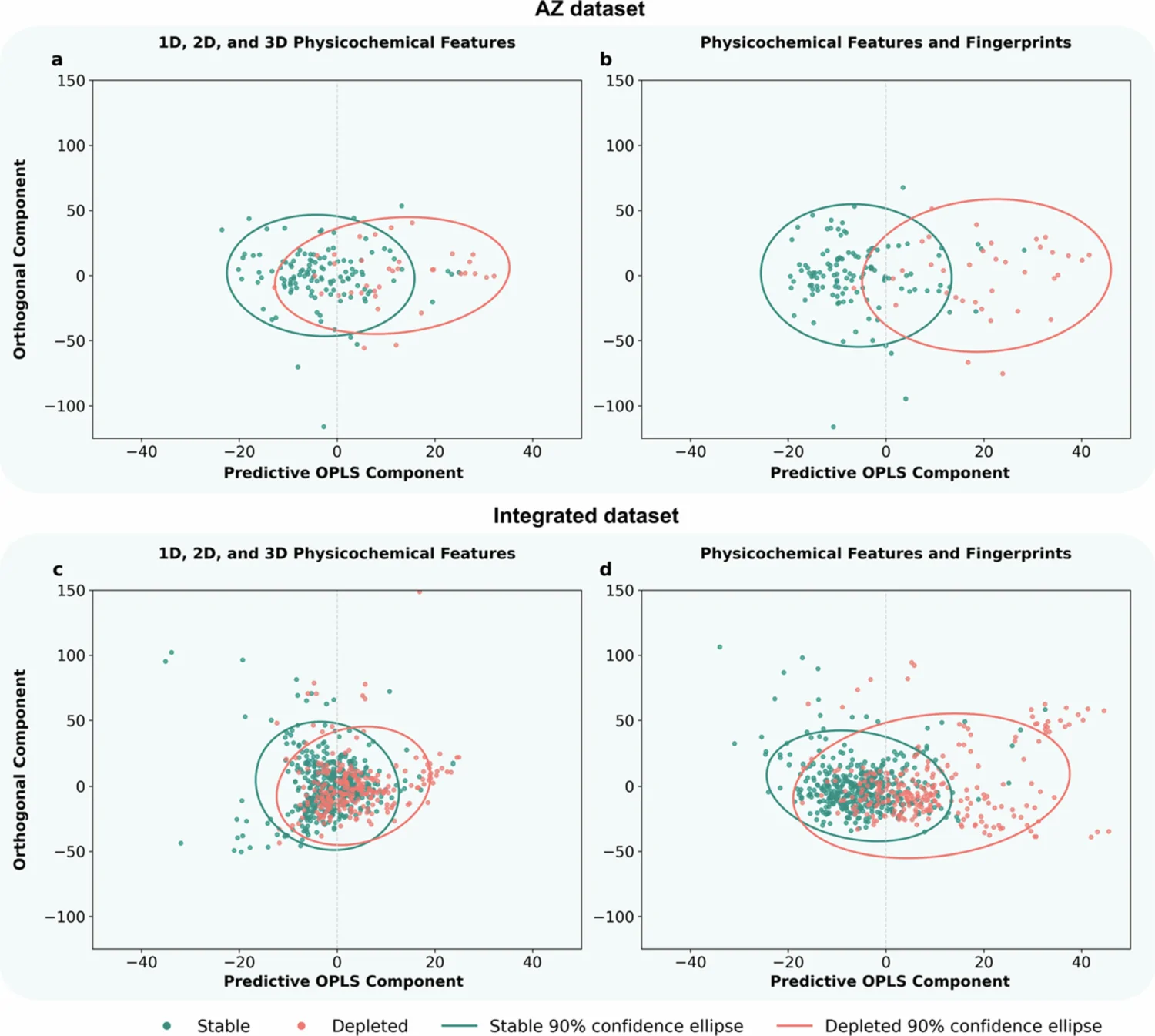
Orthogonal partial least squares (OPLS) analysis of the AZ and Integrated datasets. Panels **a** and **b** show OPLS projections of the AZ dataset using physicochemical descriptors alone (**a**) and physicochemical descriptors with fingerprints (**b**). Panels **c** and **d** show the corresponding projections for the Integrated dataset using physicochemical descriptors alone (**c**) and physicochemical descriptors with fingerprints (**d**). Stable and depleted compounds are shown as separate groups. The horizontal axis shows the first predictive OPLS component, t[1], used to assess class separation.

In the AZ dataset with physicochemical descriptors (Figure 3a), depleted compounds were predominantly distributed on the positive side of the first OPLS predictive component, t[1], whereas stable compounds clustered closer to the origin. Class separation became more distinct when fingerprints were included (Figure 3b), with 35 of 36 depleted compounds located on the positive side of t[1]. The physicochemical and combined feature models explained 8.17% and 3.29% of the predictive variance, respectively, based on R2X[1], with corresponding orthogonal variance values of 29.6% and 9.97% based on R2Xo[1].

In contrast, the Integrated dataset showed broader dispersion in both feature spaces (Figure 3c and d). Depleted compounds still tended to occupy the positive side of t[1], but class separation was less pronounced than in the AZ dataset. The predictive variance, R2X[1], was 3.44% using physicochemical descriptors and 2.02% when fingerprints were included. The corresponding orthogonal variance values, R2Xo[1], were 30.2% and 6.0%, respectively, indicating greater variation unrelated to the depletion label.

Together, these findings show that supervised OPLS revealed modest class separation that was not evident in PCA, particularly in the more experimentally consistent AZ dataset. The weaker separation observed in the Integrated dataset likely reflects greater structural and experimental heterogeneity. These results support the need for supervised machine learning models capable of capturing depletion-related patterns beyond unsupervised linear projections.

### 3.4. Supervised learning

Five supervised machine learning models were trained and compared across the four modeling scenarios described in the Methods section: logistic regression, balanced random forest, support vector machine, LightGBM, and XGBoost. This design allowed model performance to be compared across linear, kernel-based, tree-based, and gradient-boosting classifiers, and across independent dataset and feature scenarios. LightGBM is abbreviated as GBM in Figure 4 for compact labeling. Model performance was evaluated using stratified 10-fold cross-validation, with balanced accuracy, F1 score, and AUPRC used as primary performance metrics (Figure 4).

In the AZ dataset, logistic regression achieved the highest balanced accuracy and F1 score among the five models (Figure 4a and b). In the Integrated dataset, balanced random forest achieved the strongest overall performance profile, including the highest F1 score and AUPRC in the relevant feature scenarios (Figure 4c and d). These trends reflect differences in dataset structure and experimental consistency. The controlled AZ dataset supported strong performance from a linear classifier, suggesting that depletion-related patterns were captured effectively by selected physicochemical descriptors. In contrast, the Integrated dataset combined data from multiple sources, introducing greater chemical and experimental heterogeneity. This broader variability was better handled by a balanced random forest, which captures non-linear relationships and feature interactions while accounting for class imbalance.

Related tools, including DrugBug and GutBug, predict the gut bacterial enzymes, reaction classes, or bacterial species involved in xenobiotic biotransformation, whereas BiotaPredictor predicts experimentally observed compound depletion or stability under human intestinal microbiota exposure; therefore, direct numerical benchmarking was not possible because the prediction endpoints differ.^34^ ^,^ ^35^ BiotaPredictor provides the opportunity to screen small molecule drugs for microbial depletion during early development.

Although performance differences between models were not statistically significant, the best-performing model differed between datasets. Logistic regression was selected for subsequent AZ dataset analyzes because it achieved the highest balanced accuracy and F1 score. Balanced random forest was selected for subsequent Integrated dataset analyzes because it achieved the strongest overall performance across the primary metrics, including F1 score and AUPRC. These selections reflected the overall performance profile of each model within its respective dataset and feature space.

**Figure 4.**
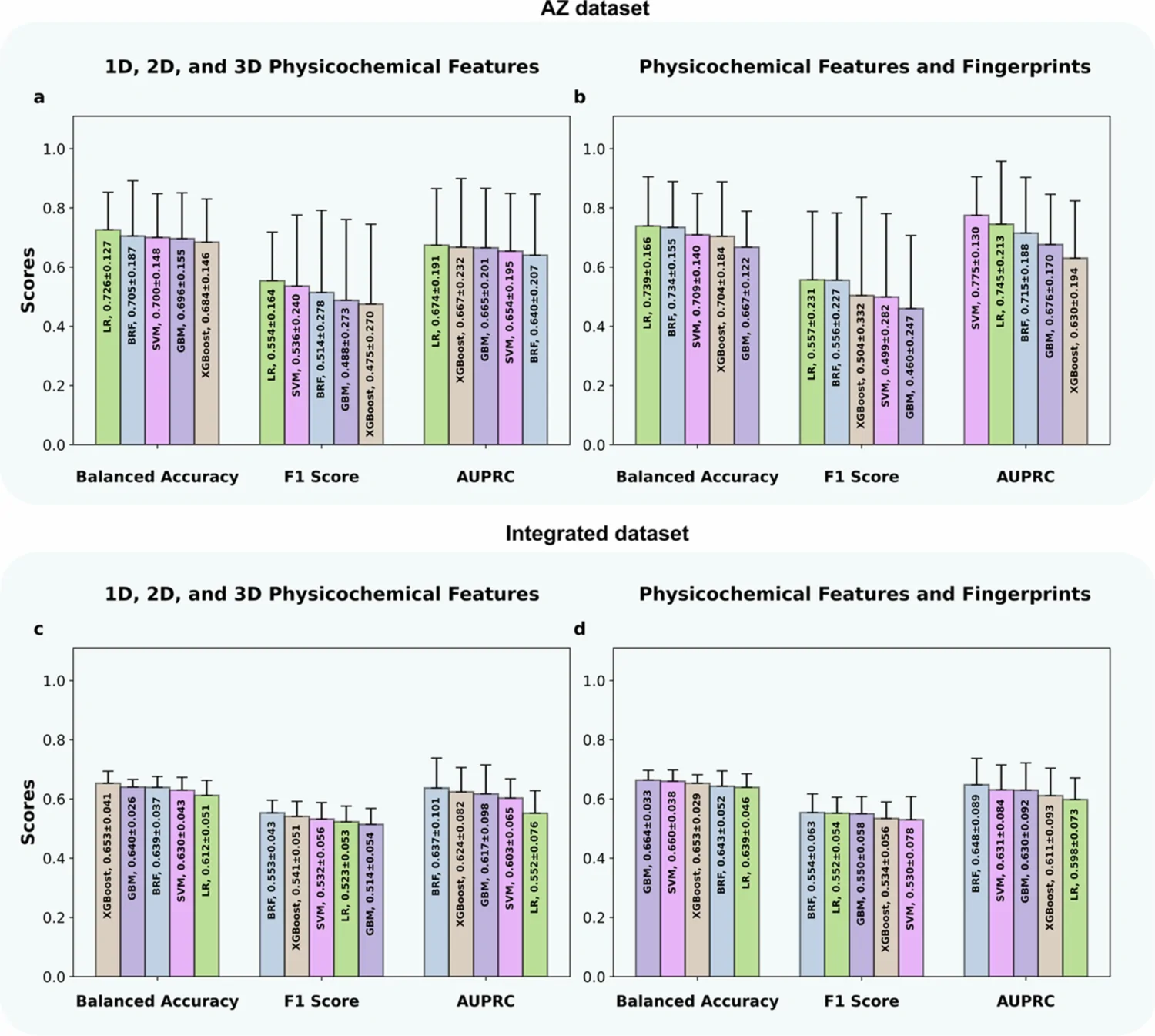
Ranked predictive performance of machine learning models on the AZ and Integrated datasets. Panels **a** and **b** show balanced accuracy, F1 score, and area under the precision–recall curve (AUPRC) for five supervised learning models trained on the AZ dataset using stratified 10-fold cross-validation: logistic regression (LR), balanced random forest (BRF), support vector machine (SVM), LightGBM, abbreviated as GBM in the figure, and XGBoost. Panels **c** and **d** show the corresponding results for the Integrated dataset. Performance metrics were calculated with depleted compounds defined as the positive class. Statistical comparisons were performed using one-way ANOVA followed by Tukey post hoc testing; no significant differences were observed between models.

### 3.5. Feature selection

Recursive feature elimination (RFE) was applied to reduce model complexity and identify informative molecular features for the selected classifiers. Logistic regression was used for feature selection in the AZ dataset, whereas balanced random forest was used for the Integrated dataset, consistent with the model selection results described above. RFE was used to reduce overfitting risk and retain features most relevant to microbial depletion, particularly in the context of limited sample size and class imbalance.^21^ The number of selected features was constrained to no more than half the number of compounds in each modeling scenario.

In the AZ dataset, feature selection substantially improved model performance, particularly when using physicochemical descriptors (Figure S3a, b). Balanced accuracy increased from 0.726 ± 0.127 to 0.938 ± 0.063, F1 score increased from 0.554 ± 0.164 to 0.879 ± 0.105, and AUPRC increased from 0.674 ± 0.191 to 0.921 ± 0.106. These improvements were statistically significant across all three metrics, indicating that feature selection enhanced predictive performance in the controlled AZ dataset. In contrast, feature selection produced more modest changes in the Integrated dataset (Figure S3c, d). Although AUPRC and F1 score increased numerically, these differences were not statistically significant. This pattern reflects the greater chemical and experimental heterogeneity of the Integrated dataset, where a smaller globally informative feature subset is more difficult to define.

To support interpretation, the most influential features from each model and dataset combination are shown in Figures S4–S7 and grouped by descriptor dimensionality. Several RFE-selected features also ranked among the highest features in the Spearman correlation analysis (Figure 2g–j). In the integrated dataset with fingerprints, PubChemFP454, PubChemFP423, and PubChemFP593 were among the top-ranked features. These fingerprints represent substructural motifs associated with amide, nitroso, nitro, or related nitrogen- and oxygen-containing functionalities, which are chemically plausible contributors to microbiota-mediated depletion through hydrolysis or anaerobic reduction, as discussed in Section 3.2.2.

Among the physicochemical descriptors, naaO, representing the number of aliphatic ether oxygens, was the only feature shared between the RFE-selected and Spearman-ranked lists in the AZ dataset using physicochemical features. This overlap is notable because the AZ physicochemical model showed the strongest external validation performance (Section 3.7). Aliphatic ether oxygens occur in solubilising and polarity-modifying groups, and their selection suggests a link between oxygen-containing functional groups and microbiota-mediated stability under anaerobic conditions. In the integrated dataset, shared features such as BCUTp-1l, GATS1s, and TDB10s reflected complementary information on electronic properties, molecular topology, and three-dimensional spatial distribution. The recurrence of these descriptors across correlation-based and model-based analyzes supports their value as indicators of microbial depletion risk.

### 3.6. Hyperparameter tuning

Hyperparameter tuning was performed for the selected classifiers using the features retained after RFE: logistic regression for the AZ dataset and balanced random forest for the integrated dataset (Figure S8). The performance changes after tuning were modest and not statistically significant, indicating that most performance gains were achieved through model selection and feature selection rather than final hyperparameter refinement.

In the AZ dataset, tuning the logistic regression model with selected physicochemical descriptors produced small numerical increases in balanced accuracy from 0.938 to 0.942 and F1 score from 0.879 to 0.890, while AUPRC remained unchanged at 0.921 (Figure S8a). In the Integrated dataset, tuning the balanced random forest model with selected physicochemical descriptors produced small numerical increases in balanced accuracy from 0.673 to 0.688, F1 score from 0.571 to 0.597, and AUPRC from 0.670 to 0.690 (Figure S8c). These changes were minor and did not reach statistical significance.

### 3.7. Experimental validation of the developed models on selected compounds

Figure 5a shows experimental depletion profiles for five representative compounds from the external assessment set. Ozanimod and AZ1 showed consistent depletion across replicates, with concentrations falling below the AZ stability threshold. In contrast, AZ2 and AZ3 maintained concentrations above the threshold over time, supporting classification as microbiota-stable under the tested conditions. AZ4 showed borderline behavior, with replicate profiles closer to the stability threshold. These experimental profiles were compared with predictions from the four final models (M1–M4). Among the five representative compounds shown, the AZ physicochemical model (M1) and the Integrated model using physicochemical descriptors and fingerprints (M4) showed agreement with the experimental classifications, with M1 also producing higher prediction confidence.

Figure 5b summarizes the external assessment results for the four models using confusion matrices and associated metrics, including balanced accuracy, F1 score, and AUPRC. This external assessment was independent of model development, and the validation compounds were not used during model training, feature selection, or hyperparameter tuning. The only compound overlapping with the integrated dataset was excluded from the M3 and M4 evaluations. The AZ physicochemical model (M1) showed complete agreement with the experimental classifications for the 12 tested compounds, including three depleted and nine stable compounds. This corresponded to balanced accuracy, F1 score, and AUPRC values of 1.00 in this limited assessment set. Because the external set was small and class imbalanced, these values should be interpreted as preliminary evidence of model utility rather than definitive proof of generalizability. Larger and more chemically diverse external datasets are required to further evaluate model robustness. Because BiotaPredictor predicts intrinsic microbiota-mediated depletion rather than systemic exposure, external validation was performed using matched anaerobic depletion experiments. Future translational validation should combine model-selected high- and low-risk candidates with mechanistic absorption or pharmacokinetic studies to determine depletion rate, and when predicted microbial depletion may translate into altered systemic exposure.

In comparison, the AZ model incorporating fingerprints (M2) classified 8 of 12 compounds in agreement with the experimental classifications, corresponding to balanced accuracy = 0.67, F1 = 0.50, and AUPRC = 0.64. The integrated dataset models showed variable external performance. M3 achieved balanced accuracy = 0.60, F1 = 0.40, and AUPRC = 0.66, whereas M4 showed stronger agreement with experimental classifications, with balanced accuracy = 0.83, F1 = 0.80, and AUPRC = 0.78.

Overall, these results indicate that physicochemical descriptors derived from a standardized anaerobic microbiota stability dataset provided the strongest external performance in this assessment. This finding highlights the value of experimentally consistent training data for early prediction of microbiota-mediated intestinal drug loss, while also underscoring the need for broader external validation before general application across wider chemical space. Together, the use of stratified cross-validation, leakage-controlled preprocessing, constrained feature selection, hyperparameter tuning, and independent experimental assessment provides multiple levels of evidence supporting the reliability of the selected models.

**Figure 5.**
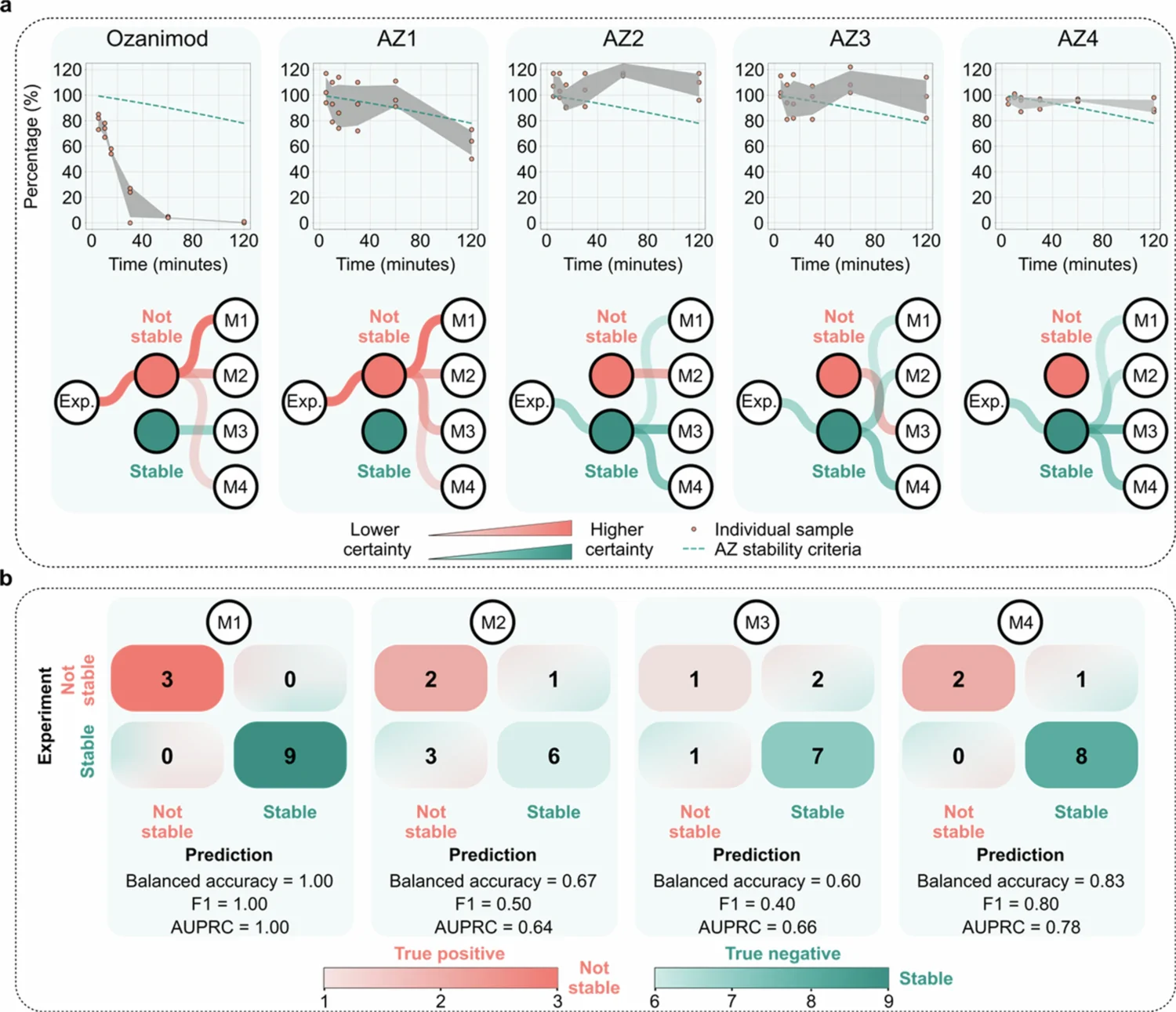
External experimental assessment of model predictions using anaerobic in vitro depletion assays. Twelve compounds, including ozanimod, methotrexate, tacrolimus, and nine internal AZ compounds, were tested in the AZ anaerobic in vitro incubation system. Panel **a** shows experimental stability profiles for five representative compounds, with corresponding model prediction outcomes shown below each profile. Markers represent individual experimental replicates, and shaded areas indicate standard deviation. The dashed line indicates the AZ internal depletion threshold. Prediction paths compare the experimental classification with the four final models, M1 to M4, with color intensity indicating prediction confidence. Panel **b** summarizes model performance using confusion matrices and associated classification metrics, including balanced accuracy, F1 score, and AUPRC. F1 score and AUPRC were calculated with depleted compounds defined as the positive class. M1 to M4 refer to models trained on: M1, AZ dataset with physicochemical descriptors; M2, AZ dataset with physicochemical descriptors and fingerprints; M3, Integrated dataset with physicochemical descriptors; and M4, Integrated dataset with physicochemical descriptors and fingerprints. One compound was excluded from M3 and M4 evaluation because it overlapped with the training data.

## 4. Conclusion

This study establishes a machine learning framework for predicting gut microbiota-mediated drug depletion as an early screening approach for oral drug delivery and formulation development. By comparing a standardized AZ anaerobic fecal incubation dataset with a larger literature-integrated dataset, we showed that experimental consistency and label harmonization strongly influence predictive performance. Logistic regression performed best for the controlled AZ dataset, whereas balanced random forest better handled the broader chemical and experimental heterogeneity of the integrated dataset. Models based on physicochemical descriptors alone and models incorporating molecular fingerprints were optimized to evaluate the contribution of general molecular properties and substructural information. To support practical use, the final models were implemented in BiotaPredictor (https://biotapredictor.onrender.com/), an openly available web-based tool for assessing microbiota-mediated intestinal drug loss during early pharmaceutical development.

The comparative analyzes emphasized the importance of standardized experimental data and consistent labeling for predictive modeling of microbiota-mediated depletion. The AZ-based models generally showed stronger performance than models trained on the more heterogeneous Integrated dataset, supporting the value of controlled anaerobic microbiota stability data for early screening. Molecular descriptors derived from energy-minimized conformers expanded the feature representation beyond simple physicochemical properties, while fingerprint features captured substructural motifs associated with microbial degradation. Together, these descriptor classes provided complementary information on molecular properties relevant to microbiota-mediated intestinal drug stability.

To assess practical model utility, we conducted anaerobic in vitro colonic incubation experiments for 12 selected compounds, including three marketed drugs, ozanimod, methotrexate, and tacrolimus, and nine internal AZ compounds. Each compound was incubated for 2 h in triplicate and classified as stable when the mean concentration remained above the established AZ stability threshold. Among the evaluated models, the AZ physicochemical model (M1) showed complete agreement with the experimental classifications, identifying all three depleted compounds and all nine stable compounds in this limited external assessment set. This corresponded to balanced accuracy, F1 score, and AUPRC values of 1.00.

These findings underscore the value of rigorous microbiota stability data, consistent labeling, and careful feature selection for developing predictive models of microbiota-mediated drug depletion. Although the present datasets are sufficient to establish and externally assess the proof-of-concept framework, they are not exhaustive. BiotaPredictor is therefore intended as an expandable platform that can be updated as additional standardized microbiota depletion datasets become available. The external assessment supports the practical potential of this framework for early candidate triage, biopharmaceutics risk assessment, and oral formulation development, including extended and delayed release strategies where distal intestinal exposure is most relevant. Although the external assessment set was limited in size, the results provide a foundation for incorporating microbiota-mediated depletion into early drug development workflows and highlight the need for broader validation across chemically diverse oral drug candidates.

## Data Availability

The source code, trained models, and supplementary scripts are available at: https://github.com/benyamin-naranjani/biotapredictor. The BiotaPredictor web tool is available at: https://biotapredictor.onrender.com/. Publicly available literature data are cited in the manuscript. Internally generated AstraZeneca depletion data are confidential and cannot be publicly shared.
